# Androgen Deprivation Therapy and Cardiovascular Risk in Chinese Patients with Nonmetastatic Carcinoma of Prostate

**DOI:** 10.1155/2014/529468

**Published:** 2014-04-07

**Authors:** Gang Huang, Chun-Yip Yeung, Ka Kui Lee, Jianxiong Liu, Kwan Lun Ho, Ming-Kwong Yiu, Karen Siu-Ling Lam, Hung-Fat Tse, Thomas Yau, Chung-Wah Siu

**Affiliations:** ^1^Division of Cardiology, Department of Medicine, Li Ka Shing Faculty of Medicine, The University of Hong Kong, Hong Kong; ^2^Cardiology Department, The Second People's Hospital of Chengdu, 610017 Chengdu, China; ^3^Division of Endocrinology, Department of Medicine, Li Ka Shing Faculty of Medicine, The University of Hong Kong, Hong Kong; ^4^Division of Urology, Department of Surgery, Li Ka Shing Faculty of Medicine, The University of Hong Kong, Hong Kong; ^5^Division of Medical Oncology, Department of Medicine, Li Ka Shing Faculty of Medicine, The University of Hong Kong, Hong Kong

## Abstract

*Background*. Androgen deprivation therapy (ADT) in nonmetastatic prostate cancer is unclear. Recent data suggests possible increase in the cardiovascular risks receiving ADT. The aim of the study was to investigate the cardiovascular outcomes in a cohort of Chinese nonmetastatic prostate cancer patients with no previously documented cardiovascular disease. *Methods and Results*. 745 patients with no previously documented cardiovascular disease and/or diabetes mellitus diagnosed to have nonmetastatic prostate cancer were recruited. Of these, 517 patients received ADT and the remaining 228 did not. After a mean follow-up of 5.3 years, 60 patients developed primary composite endpoint including (1) coronary artery disease, (2) congestive heart failure, and (3) ischemic stroke. Higher proportion of patients on ADT (51 patients, 9.9%) developed composite endpoint compared with those not on ADT (9 patients, 3.9%) with hazard ratio (HR) of 2.06 (95% confidence interval (CI): 1.03–3.24, *P* = 0.04). Furthermore, Cox regression analysis revealed that only the use of ADT (HR: 2.1, 95% CI: 1.03–4.25, *P* = 0.04) and hypertension (HR: 2.0, 95% CI: 1.21–3.33, *P* < 0.01) were independent predictors for primary composite endpoint. *Conclusion*. ADT in Chinese patients with nonmetastatic prostate cancer with no previously documented cardiovascular disease was associated with subsequent development of cardiovascular events.

## 1. Introduction

Carcinoma of prostate is the most common malignancy in men and the incidence has been increasing during the past two decades [1–3]. Androgen deprivation therapy (ADT) in form of bilateral orchiectomy and/or medical castration has been the mainstay systemic treatment in patients with metastatic disease because of the androgen dependence of the disease [1]. On the other hand, the role of ADT in localized disease remains to be defined, as many patients with localized disease often have a favorable prognosis and may live up to a near-normal lifespan. Thus, treatments aiming to further improve the long-term outcomes in these patients should outweigh any potential treatment-associated adverse effects. In recent years, the metabolic effects as well as the cardiovascular consequences of ADT had been increasingly recognized. However, clinical data concerning ADT-related adverse metabolic and cardiovascular effects remain conflicting [4]. There have been studies demonstrating that patients receiving ADT may have a higher incidence of diabetes mellitus [5–7], coronary artery disease [5, 6], stroke [6, 7], and/or even cardiovascular death [8–10], but other studies failed to show such associations [7, 11, 12]. This may be due to the fact that, in these studies, the presence of preexisting metabolic condition and other cardiovascular diseases had not been adequately characterized and/or controlled. Furthermore, previous studies often include a rather heterogeneous population from patients with distant metastasis, in whom ADT has convincingly been demonstrated to have survival benefits [13], to those with early disease in whom treatments may not be necessary. The aim of the present study is to investigate the cardiovascular outcomes in a cohort of Chinese patients with nonmetastatic cancer of prostate with no previously documented diabetes mellitus and cardiovascular disease.

## 2. Methods

### 2.1. Patients

Between January 1998 and September 2011, 1,116 Chinese patients with a diagnosis of carcinoma of prostate at Queen Mary Hospital, Hong Kong, were identified through the computer-based clinical management system. Patients were excluded if they had distant metastasis at the time of diagnosis, previously documented diabetes mellitus, coronary artery disease, congestive heart failure, ischemic stroke, chronic renal failure, and/or atrial fibrillation. As a result, the final analysis included 745 patients with nonmetastatic carcinoma of prostate, and they were then categorized according to the use of androgen deprivation therapy.

### 2.2. Study Design

This was a single-centered observational study. Data pertaining to the index carcinoma of prostate, demographics, cardiovascular risk factors, and medications were entered into the Clinical Management System Database. The primary endpoint was a composite of new occurrence of coronary artery disease, congestive heart failure, and ischemic stroke during the follow-up period. All the patients were followed up in our outpatient clinic. Deaths within the follow-up period after the recruitment were retrieved from the medical records and discharge summaries from our hospital as well as other institutions. Patients who failed to attend the clinic were contacted by phone. In addition, survival data were obtained from the Births and Deaths General Register Office.

### 2.3. Statistical Analysis

Continuous variables are expressed as mean ± standard error of mean. Statistical comparisons were performed using Student's *t*-test or Fisher's exact test, as appropriate. Kaplan-Meier survival analysis with the log-rank test was used to calculate cumulative incidences of coronary artery disease, congestive heart failure, and ischemic stroke. Multivariate analyses were performed with an enter regression model in which each variable with a *P* value ≤0.1 (based on the univariate analysis) was entered into the model. Calculations were performed using SPSS software (version 12.0). A *P* value <0.05 was considered statistically significant.

## 3. Results

A total of 745 patients (mean age: 72.2 ± 0.3 years) with nonmetastatic carcinoma of prostate were recruited, of whom 517 patients (69.3%) received ADT and the remaining 228 patients (30.7%) did not. Amongst patients receiving ADT, nearly half of them (48.7%) received medical ADT, 24.7% received surgical ADT, and 26.6% received both medical and surgical ADT.  Table 1 summarizes the baseline clinical characteristics of the study population. Patients on ADT were older (73.5 ± 0.4 years versus 69.2 ± 0.6 years, *P* < 0.01) and had a higher prevalence of being a smoker (41.4% versus 30.7%, *P* < 0.01), but a lower prevalence of hypercholesterolemia (8.1% versus 16.2%, *P* < 0.01) compared with patients not on ADT (Table 1). Concerning the tumor status, patients on ADT had a higher serum level of prostate specific antigen (430 ± 59 ng/mL versus 93 ± 44 ng/mL, *P* < 0.01), had a higher Gleason score, and are less likely to receive local treatment such as radical prostatectomy and/or radiotherapy (56.3% versus85.5%, *P* < 0.01) (Table 1).

**Figure 1:**
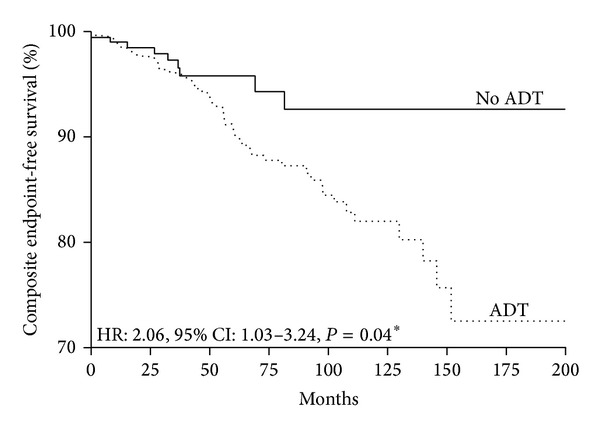
Kaplan-Meier curve of primary composite endpoint (coronary artery disease, congestive heart failure, and ischemic stroke) in patients on ADT and those not on ADT.

After a mean follow-up of 5.3 ± 0.1 years, 60 patients (8.1%) developed the primary composite endpoint (coronary artery disease, congestive heart failure, and ischemic stroke) with an annual incidence of 16.0 events per 1,000 patient-years. Of these, 51 patients were on ADT (9.9%; annual incidence: 18.6 per 1,000 patient-years) and the remaining 9 patients were not (3.9%; annual incidence: 8.9 per 1,000 patient-years).  Figure 1 depicts the Kaplan-Meier composite endpoint-free survival in patients on ADT and not on ADT. Patients on ADT had a significantly higher risk in developing the primary composite endpoint during follow-up period than those not on ADT (hazard ratio (HR) 2.06; 95% confidence interval (CI): 1.03–3.24, *P* = 0.04). In order to identify independent risk factors predicting the occurrence of primary composite endpoint, patients were categorized into (1) those with primary endpoint (*n* = 60) and (2) those without primary endpoint (*n* = 685).  Table 2 summarizes the clinical characteristics of patients with and without primary composite endpoint. Patients with primary composite endpoint had higher proportions of hypertension (53.5% versus 35.6%, *P* < 0.01) and receiving ADT (85.0% versus 68.0%, *P* < 0.01). However, there were no statistically significant differences in age and other cardiovascular risk factors, as well as tumor status (serum PSA level and Gleason score). Cox regression analysis revealed that only ADT (HR: 2.1, 95% CI: 1.03–4.25, *P* = 0.04) and hypertension (HR: 2.0, 95% CI: 1.21–3.33, *P* < 0.01) were independent predictors for primary composite endpoints.

In the analysis of the subcomponents of the primary endpoint, there were 30 newly diagnosed with coronary artery disease (7.7 per 1,000 patient-years), 14 with congestive heart failure (3.5 per 1,000 patient-years), and 21 with ischemic strokes (5.4 per 1,000 patient-years). For newly diagnosed coronary artery disease, 27 out of 30 occurred in patients on ADT (5.2%, 9.9 per 1,000 patient-years) and 3 in those not on ADT (1.3%, 2.6 per 1,000 patient-year). For patients on ADT, the proportion of patients developed coronary artery disease during follow-up period was significantly higher than that not on ADT with hazard ratio of 3.70 (95% confidence interval (CI): 1.15–5.57, *P* = 0.02). Likewise, patients on ADT were more likely to develop CHF; all 14 newly diagnosed with CHF in fact occurred in patients on ADT (2.7% (4.9 per 1,000 patient-year) versus0% (0 per 1,000 patient-year), *P* = 0.04). However, there was no statistically significant difference in the ischemic stroke risk between patients on ADT (2.9%, 5.2 per 1,000 patient-year) and those not on ADT (2.6%, 5.9 per 1,000 patient-year) (HR: 0.94, 95% CI: 0.35–2.45, *P* = 0.89).

## 4. Discussion

In the present study, we sought to investigate the effects of ADT on cardiovascular outcomes in a cohort of Chinese patients with nonmetastatic carcinoma of prostate but without previously documented cardiovascular disease. We showed that patients with nonmetastatic carcinoma of prostate treated with ADT had higher incidences of cardiovascular events including newly diagnosed coronary artery disease and congestive heart failure. Cox regression analysis identified hypertension and ADT as the only independent predictors of cardiovascular events in Chinese patients with nonmetastatic carcinoma of prostate.

Carcinoma of prostate is the most common malignancy in men with a lifetime risk of 1 in 6. In addition to local therapies, such as surgery and radiotherapy, ADT is an important systemic treatment because of the androgen dependence of the disease [1]. However, concerns about cardiovascular safety of ADT have been raised after the publication of a large observational study using Medicare database demonstrating the association between ADT and diabetes mellitus, coronary artery disease, and sudden cardiac death [5]. Subsequent observational studies likewise have demonstrated that patients with carcinoma of prostate receiving ADT had a higher incidence of coronary artery disease [6], myocardial infarct [6], sudden cardiac death [6, 9], and overall cardiovascular mortality [9, 10]. In a stark contrast, data from randomized control trials comparing ADT with placebo have failed to demonstrate any increase risk in cardiovascular events associated with ADT [11, 12, 14–22]. In a recent meta-analysis summarizing eight randomized control trials involving altogether 4,141 patients with high-risk carcinoma of prostate randomized to receive ADT or placebo, ADT has not been shown to be associated with any significant risk of cardiovascular mortality [4]. One plausible explanation for such discrepancy between randomized control trials and real-world observational registries may be related to the differences in studied subjects in these two settings. It has been postulated that as randomized control trials tend to recruit mainly healthy subjects, thus vulnerable patients with preexisting cardiovascular comorbidities and/or diseases actually experiencing excess cardiovascular events from ADT were most likely underrepresented in these randomized trials [23]. By contrast, real-world registries often include most if not all patients within a predefined geographic location, thereby representing a more diverse population of patients irrespective to their disease status and preexisting cardiovascular diseases. Unfortunately, none of the randomized control trials prestratified patients according to their baseline cardiovascular comorbidities [11, 12, 14–22]. Furthermore, information concerning preexisting cardiovascular disease was not commonly provided in detail in both published randomized control trials and observational registries; thereby it remains difficult to ascertain such explanation for the discrepancy.

In the present study, patients with carcinoma of prostate receiving ADT had higher incidences of newly diagnosed coronary artery disease and congestive heart failure. Although such findings were qualitatively consistent with previously reported observational registries that patients with carcinoma of prostate receiving ADT had significantly higher risk of subsequent cardiovascular events compared with those not on ADT [5, 6, 9, 10], there are nonetheless important quantitative differences. For instance, the incidence of newly diagnosed coronary artery disease among patients on ADT ranged from 63.3 to 72.3 per 1,000 patient-years in the very first publication raising the possible adverse cardiovascular effects of ADT involving 73,196 patients with carcinoma of prostate [5], which is 6-7 times higher than that of our cohort (only 9.9% per 1,000 patient-years). Similarly, the incidence of newly diagnosed coronary artery disease amongst patients not receiving ADT in the same study was likewise much higher than the present cohort (61.3 versus 2.6 per 1,000 patient-years) [5]. This may somehow reflect differences in baseline cardiovascular risk between studies but may also imply the baseline cardiovascular risk undermining the vulnerability to subsequent cardiovascular events from ADT. Indeed, in a retrospective cohort of 5,077 patients with localized carcinoma of prostate that patients receiving ADT had a higher all-cause mortality, such risk increased from a HR of 1.3 amongst patients with no comorbidity to 1.4 amongst those with diabetes mellitus, hypertension, and/or hypercholesterolemia and to 2.33 amongst those with preexisting congestive heart failure and/or myocardial infarction [24]. In the present study, we excluded not only patients with preexisting diabetes mellitus and/or coronary artery disease as the previously captioned study [5] but also patients with other preexisting conditions such as congestive heart failure, ischemic stroke, chronic renal failure, and atrial fibrillation, thereby representing patients with a very low cardiovascular risk. Despite such low risk, the use of ADT was associated with increased risk of cardiovascular events as in previously reported cohorts at much higher risk. Counterintuitively, a recent study by Vigen and colleagues involving 8,709 men noted to have a total testosterone <300 ng/dL after coronary angiography showed that individuals receiving testosterone therapy had a higher incidence of composite events of myocardial infarctions and strokes [25]. While the underlying mechanisms remain elusive, it has been postulated that the nonphysiologic peak and trough levels of injectable form of testosterone replacement may have contributed to the development of cardiovascular diseases/events. It is possible that, like other physiological phenomena, the association between testosterone level and the development of cardiovascular diseases/events exhibits a J-curve relationship. Taken collectively, it appears that ADT confers a higher risk of cardiovascular diseases to patients with carcinoma of prostate and the magnitude of such risk may depend on the underlying cardiovascular risk profile of individual patients. This in fact concurs with the jointed recommendation from the American Heart Association, the American Cancer Society, and the American Urological Association advocating periodic cardiovascular risk surveillance for patients with carcinoma of prostate on ADT [26].

*Limitations*. The study has several limitations. First, this study is limited by the relative small sample size and being a single-centered observational design. Second, given the relatively low-risk population, the event rate was very low, thus limiting the statistical power of the analyses of cause-specific mortality. Third, due to lack of access to the details of nature of ADT, the impact of the types as well as duration of ADT on the development of cardiovascular diseases has not been analyzed. Fourth, despite the multivariate regression model demonstrating the association between ADT and the primary composite endpoint (coronary artery disease, congestive heart failure, and ischemic stroke), it is noteworthy that patients on ADT were significantly older and possessed higher prevalence of various cardiovascular risk factors than those not on ADT. Finally, newer risk stratifiers such as high sensitive C-reactive protein have not been assessed routinely. This study nonetheless demonstrates that, even in relatively low cardiovascular risk patients with nonmetastatic carcinoma of prostate, ADT was associated with significant higher cardiovascular event rate. Further studies are needed to define the recommendations for risk stratification and therapy in this group of patients.

## Figures and Tables

**Table 1:** Baseline characteristics of patients with and without androgen deprivation therapy.

	All (*N* = 745)	ADT (*N* = 517)	No ADT (*N* = 228)	*P*-value
Age, years	72.2 ± 0.3	73.5 ± 0.4	69.2 ± 0.6	<0.01*
Hypertension, *n* (%)	276 (37.0)	189 (36.6)	87 (38.2)	0.68
Hypercholesterolemia, *n* (%)	79 (10.6)	42 (8.1)	37 (16.2)	<0.01*
Cigarette smoker, *n* (%)	284 (38.1)	214 (41.4)	70 (30.7)	<0.01*
Lung disease, *n* (%)	26 (3.5)	21 (4.1)	5 (2.2)	0.28
Carcinoma of prostate				
PSA at diagnosis (ng/mL)	326 ± 43	430 ± 59	93 ± 44	<0.01*
Gleason score				<0.01*
2–5, *n* (%)	40 (5.4)	25 (4.8)	15 (6.6)	
6–8, *n* (%)	481 (64.6)	291 (56.3)	190 (83.3)	
9-10, *n* (%)	141 (18.9)	131 (25.3)	10 (4.4)	
Not specified, *n* (%)	83 (11.1)	70 (13.4)	13 (5.7)	
Treatment				
Local treatment, *n* (%)	486 (65.2)	291 (56.3)	195 (85.5)	<0.01*
Surgical ADT only, *n* (%)	128 (17.2)	128 (24.7)	—	
Medical ADT only, *n* (%)	252 (33.8)	252 (48.7)	—	
Medical and Surgical ADT, *n* (%)	137 (18.4)	137 (26.6)	—	

*P < 0.05 (comparison between patients with and without ADT).

ADT: androgen deprivation therapy; PSA: prostate specific antigen.

**Table 2:** Baseline characteristics of patients with and without primary composite endpoint.

	No primary endpoint (*N* = 685)	With primary endpoint (*N* = 60)	*P* value
Age, years	72.1 ± 0.3	73.4 ± 1.5	0.29
Hypertension, *n* (%)	244 (35.6)	32 (53.5)	<0.01*
Hypercholesterolemia, *n* (%)	75 (10.9)	4 (6.7)	0.39
Cigarette smoker, *n* (%)	257 (37.5)	27 (45.0)	0.25
Lung disease, *n* (%)	24 (3.5)	2 (3.3)	1.00
Carcinoma of prostate			
PSA at diagnosis, ng/mL	348 ± 47	43 ± 13	0.07
Gleason score			0.20
2–5, *n* (%)	34 (5.0)	6 (10.0)	
6–8, *n* (%)	444 (64.8)	37 (61.7)	
9-10, *n* (%)	133 (19.4)	8 (13.3)	
Not specified, *n* (%)	74 (10.8)	9 (15.0)	
Treatment			
Local treatment, *n* (%)	452 (66.0)	34 (56.7)	0.15
ADT, *n* (%)	466 (68.0)	51 (85.0)	<0.01*
Surgical ADT only, *n* (%)	113 (16.5)	15 (25.0)	
Medical ADT only, *n* (%)	224 (32.7)	28 (46.7)	
Medical and Surgical ADT, *n* (%)	129 (18.8)	8 (13.3)	

*P < 0.05 (comparison between patients with and without ADT.

ADT: androgen deprivation therapy; PSA: prostate specific antigen.
